# Kali-bichromicum

**Published:** 1899-11

**Authors:** 


### HOMŒOPATHIC PHYSICIAN,

A MONTHLY JOURNAL OF

HOMŒOPATHIC MATERIA MEDICA AND CLINICAL MEDICINE.

"If our school ever gives up the strict inductive method of Hahnemann, we are lost, and deserve only to be mentioned as a caricature in the history of medicine."—CONSTANTINE HERING.

Vol. XIX. NOVEMBER, 1899. No. 11.

KALI-BICHROMICUM.—The frequent requests on the part of the readers of this journal for a continuation of the notes from Dr. Lippe's lectures have induced the editor to once more resume them, notwithstanding the other demands upon his time. Accordingly we begin with Kali-bichromicum. This remedy is of the greatest use in the treatment of catarrh of the nose. It is particularly useful in cases where the discharge has been suppressed by applications.

A remarkable and constant characteristic of the remedy is headache preceded by temporary blindness. The sight is restored as the headache increases. There is another characteristic of Kali-bichromicum. It is headache preceded by shimmering before the eyes like that of sunlight on the surface of a sheet of water.

Kali-bichrom. has stinging headache and Pulsatilla similarly stinging headache here and there.

This remedy has morning headache. Many remedies have this same symptom, but Dr. Lippe mentioned only four. Kali-bichrom., Nux-moschata, Nux-vomica, and Lycopodium.

Kali-bichrom. has headache from suppression of the discharge from the nose in nasal catarrh. Hence the statement previously made that it is useful in suppression of nasal catarrh.

Kali-bichromicum has heaviness of the upper eye-lid on waking; it requires an effort to open it.

Dr. Guernsey's keynote of Causticum is paralysis of the upper eye-lids.

Dr. Lippe gave the following comparisons:

Sepia, eye-lids painful on waking as if too heavy and as if he could not keep them open. Inability to open the eye-lids at night.

Rhus-tox., heaviness of the eye-lids. Swelling of eye-lids.

Natrum-muriaticum, spasmodic contraction of eye-lids.

Argent-nit., left upper eye-lid falls further over the eye than the right.

Gelsemium, heaviness of the eye-lids, cannot keep the eyes open.

Mercurius, violent contraction of eye-lids; it is difficult to open them.

Pulsatilla, the eyes are glued together in the morning preventing their being opened until washed.

Kali-bichrom. and Pulsatilla both have the eyes glued together in the morning.

Kali-bichrom. has œdematous swelling of the eye-lids, this is similar to Apis.

Kali-bichrom., edges of eye-lids seem rough, causing sensation of friction as if from sand on the eye-balls. Sensation of sharp sand in the eyes.

Arsenic has sensation of great dryness of edges of eye-lids and as if they were scratching the eye-ball. The tarsi burn.

Natrum-muriaticum, sensation of sand in the eyes in the morning.

Caulophyllum, sensation of something under the eye-lids.

Mercurius, pains under the eye-lids as if from a cutting body.

Kali-bichrom. has soreness of right caruncula.

Bromine has darting pains through left eye.

Kali-bichromicum has photophobia.

Mercurius has photophobia with violent inflammation of the eye.

Pulsatilla has photophobia with stitches in the eyes from the light.

Kali-bichromicum has lachrymation and burning of the eyes.

Mercurius has violent lachrymation in the evening.

Pulsatilla has lachrymation in the open air and in the wind.

Thuja has lachrymation in the open air. The tears do not run off but remain standing in the eye.

Kali-bichrom. has brown spots on the conjunctiva and Nux-vomica has red spots.

